# Quorum Sensing as a Trigger That Improves Characteristics of Microbial Biocatalysts

**DOI:** 10.3390/microorganisms11061395

**Published:** 2023-05-25

**Authors:** Elena Efremenko, Olga Senko, Nikolay Stepanov, Aysel Aslanli, Olga Maslova, Ilya Lyagin

**Affiliations:** Faculty of Chemistry, Lomonosov Moscow State University, Lenin Hills 1/3, 119991 Moscow, Russia

**Keywords:** quorum sensing, triggering factors, biocatalysts, yeast cells, bacteria, filamentous fungi, consortia, ATP control

## Abstract

Quorum sensing (QS) of various microorganisms (bacteria, fungi, microalgae) today attracts the attention of researchers mainly from the point of view of clarifying the biochemical basics of this general biological phenomenon, establishing chemical compounds that regulate it, and studying the mechanisms of its realization. Such information is primarily aimed at its use in solving environmental problems and the development of effective antimicrobial agents. This review is oriented on other aspects of the application of such knowledge; in particular, it discusses the role of QS in the elaboration of various prospective biocatalytic systems for different biotechnological processes carried out under aerobic and anaerobic conditions (synthesis of enzymes, polysaccharides, organic acids, etc.). Particular attention is paid to the biotechnological aspects of QS application and the use of biocatalysts, which have a heterogeneous microbial composition. The priorities of how to trigger a quorum response in immobilized cells to maintain their long-term productive and stable metabolic functioning are also discussed. There are several approaches that can be realized: increase in cell concentration, introduction of inductors for synthesis of QS-molecules, addition of QS-molecules, and provoking competition between the participants of heterogeneous biocatalysts, etc.).

## 1. Introduction

**Figure 1:**
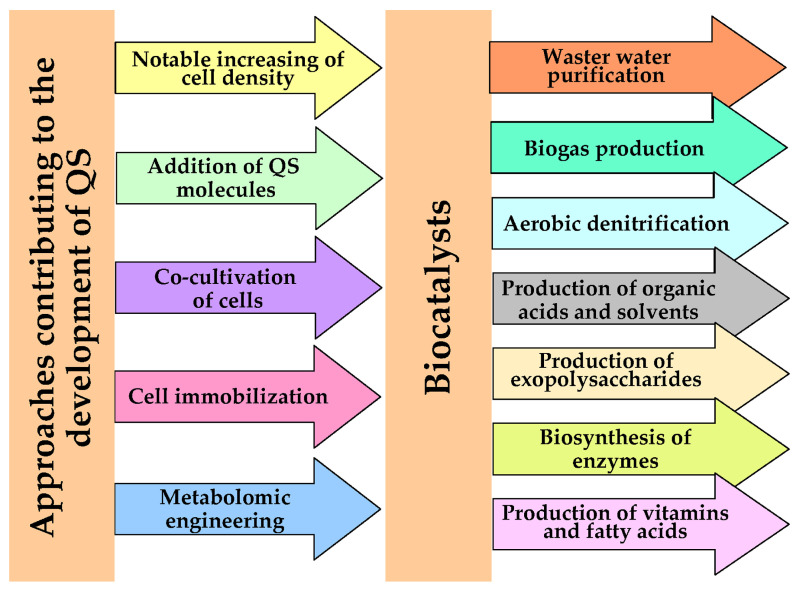
The factors used as triggers of QS as a regulator of improved properties of BCs catalyzing various biotechnological processes.

A number of biotechnological processes based on the use of highly concentrated populations of cells of various microorganisms in order to intensify them and improve the main characteristics (productivity and stability of functioning) of biotechnological processes are known today [1,2,3,4]. At the same time, highly concentrated cell populations are considered as effective biocatalysts (BCs) of various biotechnological processes that allow a variety of target products (polysaccharides, organic solvents and acids, enzymes, biogas, vitamins, etc.) to be obtained (Figure 1). The reason for the interest in such BCs lies in the manifestation by cells in such populations of properties characterized as the state of quorum sensing (QS). QS consists in changing the biochemical status of cells in comparison with single cells of the same microorganism due to the built-in genetic program, which is implemented at a certain concentration per unit volume under the influence of a total concentration of substances called quorum inducers. To date, the appearance of QS is known as a general mechanism of cell-to-cell chemical communication dependent on population density revealed for different microorganisms (bacteria, microalgae, yeast, filamentous fungi), including those that are part of complex consortia [2,3,5,6,7]. Initially (50 years ago) QS was discovered as a mechanism responsible for cellular communication in populations of Gram-negative bacteria *Vibrio fischeri* [8].

It is well known today that cells of microorganisms used in various biotechnological processes as biocatalysts and composed populations in QS state have a number of advantages over single cells [9]:

- Increased resistance to negative external influences (pH, temperature, culture components, the presence of toxins, antimicrobial substances, etc.);
- The ability to long-term preservation of metabolic activity during storage and repeated use in biotechnological processes;
- Increased total productivity of the processes in which they are used as biocatalysts due to the possibility of maintaining high concentrations of cells per unit volume in the absence of noticeable cell lysis.

Usually, one of the most important characteristics of cells of different microorganisms that transform into a state of QS is the synchronization of their functional activity [10], which leads to the simultaneous implementation of the same biochemical metabolic pathways by the majority of cells in such highly concentrated populations (“mainstreams”). This allows cells to increase the yields of certain metabolites and obtain the necessary energy reserve in the form of adenosine triphosphate (ATP) [9]. This reduces the variability (narrowing the range) of the byproducts with a simultaneous decrease in their concentrations, which contributes to the production of a biotechnological culture with fewer by-products and simplifies the separation of target low-molecular metabolites from them [10,11]. 

As the recent studies of various BCs have shown, the transition of cells from the normal state into the state of QS can be caused by the use of different approaches (Figure 1) [12,13,14,15,16,17,18,19,20,21,22,23,24,25,26,27,28,29,30,31,32,33]. The summation of these approaches and the analysis of their effectiveness have undoubted scientific and practical significance, and therefore have become the object of attention of this review. At the same time, the role of QS for the success in obtaining and application of BCs was analyzed using the example of various microorganisms. The review analyzed the results of experiments conducted by scientists mainly in the last 5 years, which made it possible to highlight the main current trends in the research itself, as well as the interest in those biotechnological processes; the development of BCs for these processes attracts the most attention today.

## 2. General Approaches to the Regulation of QS in Bacteria Used as BCs in Various Biotechnological Processes

The effect of QS induced by specific molecules of Gram-negative bacteria, *N*-acyl homoserine lactones (AHL), on the physicochemical properties of ANAMMOX granulated sludge [12,13,14,15,16] has been actively studied in recent years (Table 1) [12,13,14,15,16,17,18,19,20,21,22,23,24,25,26,27,28,29,30,31,32,33].

For the development of QS, bacteria use various signaling molecules, autoinducers (AI), which bind to cytoplasmic receptors in bacterial cells and activate the coordinated expression of target genes when the population reaches the quorum concentration. The most common signaling molecules of QS used by most Gram-negative and Gram-positive bacteria are N-acyl homoserine lactones (AHL) and modified oligopeptides (autoinducing peptides, AIP), respectively. To date, various small cyclic furanone compounds (autoinducers-2, AI-2), widely distributed among both Gram-positive and Gram-negative bacteria, are also known, which provide intra- and interspecies communication between them [1].

The Lux-type QS system (LuxR/LuxI circuit), which is mediated by AHL molecules, is common in many Gram-negative bacteria (Table 1). The LuxI and LuxR are important proteins for QS regulation of bioluminescence in *V. fischeri*, where LuxI (AHL synthase) catalyzes the synthesis of autounducer, which is then detected by transcription factor LuxR (cytoplasmic receptor). LuxR-type proteins consist of an N-terminal AHL-binding domain and a C-terminal DNA-binding domain. The binding of AHLs to LuxR results in the formation of LuxR-AHL complex, which binds “*lux* box” sequence and activates the expression of target genes [34].

Pairs of LuxR/LuxI homologues, which are critical for regulating diverse physiological functions (bioluminescence, biofilm formation, etc.), have been identified in many bacterial species. Two different homologues of Lux-type QS systems such as Las (LasR/LasI) and Rhl(RhlR/RhlI) regulate the cascade of virulence regulators in *Pseudomonas aeruginosa* [32], including exoprotease secretion, toxin production, motility, and biofilm formation [35].

There are also a small number of LuxR homologues, such as EsaR, from *Pantoea stewartii* subsp. *stewartii*, that naturally act as repressors. The cells regulate the biosynthesis of exopolysaccharides by the EsaR/EsaI QS system, where EsaI encodes AHL-synthase in the same way as other LuxI-type family proteins. The transcription factor EsaR represses or activates transcription in the absence of AHL (at low cell densities), and derepresses or deactivates transcription when combined with AHL (at high cell densities) [36].

The Gram-positive bacteria *Staphylococcus aureus* QS is encoded by the Agr (accessory gene regulator) system, which plays a crucial role in regulating the syntheses of a wide range of *S. aureus* virulence factors and complex association with biofilm formation [6,20]. The Agr QS system is composed of the response regulator protein, AgrA, and the sensor histidine kinase, AgrC, which expresses a regulatory RNA, RNAIII, in response to autoinducing peptides [37].

The QS proteins of Gram-positive bacteria (e.g., *Bacillus* sp., *Enterococcus faecalis*) consisting of certain aspartyl phosphate phosphatases (Rap proteins), the regulator of the glucosyltransferase gene (Rgg), the neutral protease regulator (NprR), the phospholipase C regulator (PlcR), and the pheromone receptor (PrgX) form a protein family named RNPP, which is characterized by the presence of the tetratricopeptide repeat. There are only a few examples of the applications of these QS systems in biotechnological processes. For example, a significant regulatory role of RNPP-type QS systems in the solvent formation, cell motility and sporulation of *Clostridium saccharoperbutylacetonicum* N1-4 cells was reported [33].

The study of the effect of a number of AHL molecules (*N*-3-oxo-hexanoyl homoserine lactone (3OC6-HSL), *N*-hexanoyl homoserine lactone (C6-HSL), *N*-octanoyl homoserine lactone (C8-HSL), and *N*-dodecanoyl homoserine lactone (C12-HSL)) on metabolic processes in ANAMMOX consortia and on the ANAMMOX activity of granular sludge biomass has made it possible to establish that all studied AHL molecules are involved in the regulation of (i) bacterial activity in ANAMMOX consortia, (ii) the synthesis of amino acids (Ala, Val, Glu, Asp, and Leu), and (iii) the pathways of *N*-acetylmannosamine (ManNAc) and uridine-diphospho-N-acetylgalactosamine (UDP-GlcNAc), promoting the production of extracellular polymeric substances. C6-HSL and C8-HSL molecules significantly affected the metabolic activity of cells and the formation of a granular biocatalyst [12,13,14]. A significant effect of the substrate shock (excess concentration of the substrate) (total nitrogen) on the level of synthesis of AHL molecules in the granules of ANAMMOX bacteria and the correlation between the concentration of AHLs (C6-HSL and C8-HSL), excretion of extracellular polymeric substances, and the characteristics of self-forming granules of the biocatalyst has been shown [15]. The study of HdtS-type AHL synthases (JqsI-1 and JqsI-2) identified in the ANAMMOX bacteria *Candidatus Jettenia caeni* revealed a positive correlation between AHL concentration, AHL synthase gene expression, and the hydrazine synthase gene hzsA (genetic marker of ANAMMOX activity) [16].

Microbial synthesis through metabolic engineering is a widely used approach to increase the production of target products in various industries. However, this requires constant regulation of the concentration of the cell population to maintain their productivity. Recently, the majority of studies have focused on the development of various strategies for the use of the components of the QS, a mechanism of interaction between microorganisms induced by various quorum sensing molecules (QSMs), for the dynamic regulation of metabolic pathways for the synthesis of target products (Table 1) [17,18,19,20].

*E. coli* cells are most often used as host cells for the production of a number of products due to the presence of thoroughly studied metabolic pathways, engineering tools, and the ease of culturing highly concentrated populations [17,18,19,20,21,22,23,24,25]. In studies [17,19,21,23], synthetic microbial biocatalysts were developed and constructed using components of the Lux-type QS system in genetic circuits to regulate metabolic pathways for the biosynthesis of isopropanol, salicylic acid, 4-hydroxycoumarin, bisabolene, alginate lyase, and esterase. A modified version of the QS-MTS (metabolic toggle switch) system to stabilize the efficiency of QS signaling and regulation of metabolism was constructed in *E. coli* cells to increase the production of pyruvate and acetate [25].

Components of the Esa-type QS system have also been used in the development of genetic circuits and have been successfully applied for dynamic regulation of the biosynthetic pathways of 4-hydroxyphenylacetic acid (4HPAA) and 5-aminolevulinic acid (ALA) in *E. coli* cells [20,24].

New expression cassettes based on the switchable feedback promoters (SFP) regulatory motif, where small RNAs are used as transcription regulators to integrate additional levels of control over transcription outputs was developed in the research [18]. Lux- and Esa-type QS systems have been used to regulate these expression cassettes (riboregulated SFP, rSFP), which have been successfully applied for the regulation of the amorphadiene and oxygenated taxane metabolic pathways. The construction of two orthogonal, autonomous, and customizable genetic circuits based on the components of Lux- and Agr-type QS systems for dynamic modulation of the expression of two different sets of enzymes has made it possible to achieve a significant increase in the yield of medium-chain fatty acids, both in cultures in flasks and in bioreactors [22].

In addition to *E. coli* cells, a number of other microorganisms, in particular bacteria of the genus *Bacillus*, *Gluconobacter*, *Pseudomonas*, etc., are also actively used as host cells in metabolic engineering [26,27,28,29,30,31,32].

The integration of two modular QS systems, Phr60-Rap60-Spo0A and PhrQ-RapQ-DegU, for the dynamic control of metabolic pathways for the synthesis of menaquinone-7 (vitamin K_2_) or γ-polyglutamic acid (γ-PGA), respectively, in *B. subtilis* 168 cells resulted in a significant increase in the level of their production [26,27]. The study [28] proposed a completely autonomous system for controlling gene expression in *B. subtilis* cells based on the components of the Lux-type QS system from Gram-negative *V. fischeri* cells. The success of such QS modification was demonstrated in the production of vitamin B_2_.

A programmed cell-death module based on the Lux-type QS system was installed in *G. oxydans* cells to optimize the process and eliminate the occurrence of competition between *G. oxydans* and *Ketogulonicigenium vulgare* in a consortium of three species (*G. oxydans—K. vulgare—Bacillus megaterium*) for one-step production of 2-keto-L-gulonic acid (2-KGA) from sorbitol [31].

The study of the possibility of regulating denitrification by *P. aeruginosa* cells under aerobic conditions using two QS systems Las and Rhl has made it possible to establish that the Las and Rhl systems suppress the expression of target genes *napA, nirS, norB, norC*, and *nosZ*, thereby negatively affecting the activity of enzymes that catalyze denitrification, namely, nitrate reductase (NAP), nitrite reductase (NIR), nitric oxide reductase (NOR), and nitrous oxide reductase (NOS) [32].

Genetic engineering techniques can also be applied to control QS. It is known that surfactin correlates with the carbon metabolism in *B. amyloliquefaciens* cells which are relative of *B. subtilis* [29]. Mutation of *srfA* (Δ*srfA*—a gene cluster for biosynthesizing surfactin) in *B. subtilis* led to enhanced production of acetate [30].

Thus, various approaches can be applied to stimulate QS in different bacterial cells by adding QSMs to the cell culture medium, metabolic engineering, and genetic modification. A natural or artificial increase in the concentration of the cells and their immobilization can also be used to improve the characteristics of biotechnological processes based on cellular BCs, and this will be discussed further.

## 3. QS in Biosynthesis of Polysaccharides by Bacterial BCs

The interest in the production of various polysaccharides (PSs) by biotechnological methods is enormous today [38]. At the same time, it was clearly shown that there is a relationship between QS, which depends on the population density of bacterial cells in the medium, and the level of exopolysaccharide synthesis [39]. Bacterial cells are the main source for obtaining active BCs that produce various PSs, since it is bacteria that have the natural ability to form biofilms that play a key role in their adhesion and protection from negative factors, including toxic and antimicrobial substances [40], as well as in the implementation of the survival strategy with a decrease in the concentration of available substrates. Various PSs (cellulose, levan and alginate) were found in the composition of bacterial biofilms [38,41].

In recent years, a growing number of studies have focused on studying the main mechanisms underlying the regulation of PS biosynthetic pathways. This is necessary for understanding the basic principles of functioning of processes and their shift towards the formation of the target product [42,43]. Most of the enzymatic steps in PS biosynthesis occur within the cells, while polymerization and secretion are localized in the cell wall. There are also examples of extracellular biosynthesis of PSs (dextran or levan). However, in both cases, the effect of QS on PS production is extremely interesting, since it can increase the efficiency of this process by activating or inhibiting key enzymes of the synthesis [42,43]. Thus, the biosynthesis of bacterial cellulose (BacC) depends on the presence of an allosteric regulator (cyclic guanosine monophosphate, c-GMP), which accumulates during the formation of biofilms, and can increase in its presence by almost 100 times. The mechanism of this regulation was revealed when the crystal structure of the cellulose synthase complex was studied and it was shown that the enzyme, being in an inactive state, interacts with c-GMP, which contributes to its activation [44].

The level of productivity of the main metabolites in the cells in biofilms is at a significantly higher level in comparison with free cells. The same general effect is observed in most cells artificially immobilized in a concentrated form (Table 2) [45,46,47,48,49,50,51,52,53,54,55,56,57,58]. It should be noted that poorly concentrated cell samples did not give improved levels of accumulated products even in immobilized form compared to the same free cells [49]. The influence of some carriers used for immobilization of cells in the QS-state and their low porosity created additional limitations for mass transfer to realize the advantages of QS; therefore, the yields of the products were comparable to those typical for free concentrated cells [57,58].

A significant increase in the biosynthesis [45,46,47,48,50,51,52,53,54,55,56] or the achievement of a concentration comparable to that accumulated by free cells in highly concentrated suspensions, which are generally formed by the end of the logarithmic growth phase [49,57,58], was shown for various PS producers as a result of their immobilization. In addition, the use of different methods of immobilization (adsorption on an insoluble carrier [48,50,51,53,54,55,56,57] or inclusion in a gel matrix [45,46,47,49,52,58]) led to the transition of bacterial cells into the QS state because the procedure resulted in a high-density cell culture and in keeping the cells in this state. The immobilization of PS producers in gel matrix has long been considered impractical due to diffusion difficulties and hampered mass transfer, which limits cellular metabolism [38]. However, the successful biosynthesis of such high molecular weight polymers as BacC, dextran, and pullulan has been proven by incorporating producer cells into PVA cryogel [45,46,47]. It was also noted that the accumulation of free cells in the medium during the cultivation of thus immobilized producers was six times less than in the case of suspension cultures, and an increase in the concentration of cells immobilized in a gel matrix led to a noticeable increase in PS synthesis [38,45,46,47].

Compared to a simple increase in the concentration of cells, the presence of the matrix intensified the biosynthesis even more, for example, of BacC by *K. xylinum* bacteria cells, which synthesized and easily pushed out the resulting BacC strands through the pores of the polymer carrier, which over time combined into a dense gel film, without covering the cells. The latter, therefore, were deprived of the ability to transform into a state of rest and even more actively carried out the synthesis of BacC [33]. A similar effect of stimulating the functioning of BacC in the QS state was demonstrated in *G. kombuchae* cells immobilized using a loofa sponge [48]. The level of BacC synthesis increased by 1.6–2.1 times with their participation, in comparison with highly concentrated suspensions of free cells.

The role of QS in BacC synthesis was confirmed when it was shown that the activation of proteins responsible for alginate synthesis depends on the presence of an allosteric regulator (cyclic dimeric guanosine monophosphate, c-di-GMP), which is responsible for the QS of cells. It turned out that the Alg8 enzyme, being in an inactive state, interacts with the c-di-GMP-binding domain PILZ of the Alg44 protein, which leads to its significant activation. Alginate biosynthesis is regulated at the transcriptional and post-translational levels [59].

It should be noted that in the case of alginate producers (*A. vinelandii*) immobilized in a composite agar layer/microporous membrane, additional stimulation of PS synthesis was shown to increase by 2.7 times per 1 g of consumed substrate (sucrose) in comparison with concentrated suspension-free cells, also in the state of QS [49]. During the synthesis of xanthan by *X. campestris* cells immobilized on polyurethane foam, an increase in its biosynthesis by 267–274% was also observed [50,51].

Such a significant increase in the yield of alginate when using immobilized BCs is explained by an even greater decrease in the consumption of consumed sucrose for bacterial growth in comparison with free cells when the cells in the carrier matrix are limited by oxygen. Interestingly, the natural self-immobilization of bacteria in biofilms also turned out to lead to “overproduction” of PSs, similar to what was obtained with artificially immobilized cultures. In this regard, the use of immobilized BCs based on PS-producing cells is of great interest for biotechnology.

However, it should be noted that despite many examples of the fact that higher yields of many target PSs can be obtained as a result of the use of immobilized cells compared to concentrated suspension cultures [45,46,47,48,50,51,52,53,54,55,56], there are examples of when such an effect is absent. In particular, this applies to PSs synthesized by lactic acid bacteria [57,58]. The production of PSs using six BCs in the form of lactic acid bacteria strains (*Lactobacillus bulgaricus, L. acidophilus, L. plantarum, L. casei, Streptococcus thermophilus*, and *Lactococcus lactis*) was investigated. The highest production of PSs was obtained in the case of *L. plantarum* cells (1513.1 mg/L). Immobilization of the cells of this strain in various carriers (Ca-alginate gel, K-carrageenan, agar, gelatin cross-linked with glutaraldehyde) made it possible to obtain only 1489.9 mg/L of PSs.

In this regard, it must be emphasized that QS for immobilized cells remains a poorly studied phenomenon. A deep understanding of all the mechanisms of possible regulation of the functioning of immobilized cells as very promising BCs will allow their large-scale use in various processes in practice to be reached faster.

## 4. Biocatalysts for Biotechnological Processes Based on Highly Concentrated Yeast Populations in Suspension and Immobilized Form

It is known that morphological transformations take place with yeast cells (formation of pseudohyphae) under the action of their QSMs when changing the density of their populations. At the same time, the cells form flocculi and sediment, forming hyphae forms that are more stable to the effects of various negative factors. It is established that farnesol, tyrosol and dodecyl alcohol are QSMs for *Candida albicans* cells. The secretion of phenylethyl alcohol and tryptophol is observed in the yeast *Saccharomyces cerevisiae* during nitrogen starvation, and these compounds regulate cell density and their morphological changes. The possibility of regulating the morphological and biochemical characteristics of different yeast populations using QSMs is currently being investigated in various biotechnological processes (Table 3) [60,61,62,63,64,65,66,67,68,69,70,71,72].

In wastewater treatment, various environmental stressors can cause changes in the morphology of yeast cells and lead to a decrease in treatment efficiency. The effect of phenylalanine on *C. tropicalis* cell density, cell flocculation, and metabolism in wastewater treatment has been investigated [60]. Phenylalanine has been used to simulate induction factors of filamentous form. It turned out that with an increase in cell density to 10^8^ CFU/mL, the utilization of phenylalanine by cells decreased, the concentrations of tyrosine in extracellular metabolites increased, and the concentration of ethanol and organic (acetic, propionic, butyric, and valeric) acids decreased. Molecules of phenylethyl alcohol were found in metabolites as QSMs, and their concentration increased with the growth of the cell population, which proves that the change in cell morphology depends on QS [60,61]. The BCs generally functioned well, and the COD removal rate reached 95.3% [61].

It should be noted that in a large-scale biotechnological process, an increase of only a few percent in the yield of ethanol is of significant value for the alcohol industry. During the fermentation process, *S. cerevisiae* converts sugars into ethanol via glycolysis, producing secondary metabolites such as glycerol, acids, alcohols, and aldehydes. The synthesis of these by-products reduces the yield of ethanol. Another reason for the increase in ethanol yield (carbon substrate conversion efficiency) is a decrease in biomass growth during fermentation so that more substrate can be converted into the desired product. According to studies [60,61,62,64], QSMs allow the regulation of the yield of ethanol. Interestingly, the addition of phenylethanol in flow bioreactor experiments [62] reduces the rate of ethanol production and glucose consumption, indicating an effect of this compound on the rate of yeast catabolism.

From the scientific and practical point of view, it is extremely interesting to study the possibility of regulating ethanol fermentation with the participation of several different yeast cultures used in the QS state [66,67,69]. It has been shown that in this case the mechanisms of the regulation of processes under the action of QSMs become significantly more complicated, the dependences on the introduced concentrations of QSMs in such biotechnological media change, and the expected effects do not have the linearity that can be expected when using individual yeast cultures as a BC [66,67,69]. Thus, the effect of tryptophol or melatonin on alcoholic fermentation was investigated under the action of a BC in the form of a mixture of *S. cerevisiae* suspension cells and four species of yeast that do not belong to the genus *Saccharomyces* (*T. delbrueckii, M. pulcherrima, H. uvarum,* and *S. bacillaris*). It was found that the addition of 0.5 g/L tryptophol to the fermentation medium resulted in the inhibition of fermentation by *S. cerevisiae* cells, which continued throughout the entire process. However, this effect disappeared in the presence of other non-*Saccharomyces* strains [67].

It should be noted that changes in the morphological characteristics of yeast cells contribute to their high adhesive strength, which is caused by the presence of glycoproteins (adhesins) on the surface of yeast cell walls [73]. These adhesins are also involved in the interactions of yeast cells with each other and with cells of other microorganisms. In particular, it is these filamentous forms of cells capable of forming biofilms that are characterized by the highest adhesiveness in the yeast *C. albicans* [74,75,76]. It turned out that the aromatic alcohols tryptopol and phenylethyl alcohol activate the QS pathway in *S. cerevisiae* cells, regulating filamentation.

Hyperfilamentation is stimulated in the same cells in response to exposure to several short-chain alcohols, including isoamyl alcohol and 1-butanol. Alcohols stimulate the appearance of cyclic AMP in yeast cells, which indirectly promotes the transcriptional activation of adhesin, which is involved in cell adhesion to each other and different surfaces and enhances yeast cell filamentation [77].

The ability of emerging hyphae to secrete adhesins promotes the maturation of yeast biofilms, providing their architectural stability and the strength of emerging self-immobilizing yeast populations [78]. In this regard, the introduction of such alcohols into a medium with an increased concentration of yeast cells contributes to their transition to a productive and stable state. In support of this, the influence of the introduction of tryptophol and 2-phenylethanol into the fermentation medium on the efficiency of the process of obtaining ethanol from hydrolysates of *Desmodesmus armatus* algae was studied. Yeast *S. cerevisiae* cells immobilized in Ca-alginate gel were used as a BC. It was shown that the addition of 2-phenylethanol at a concentration of 0.2% to the medium leads to an increase in the yield of ethanol by 7.4% [63].

Artificial immobilization of highly concentrated yeast cells promotes their transition into the QS state and keeps them in such state where the cells can be used as an effective BC for a long time (Table 4) [79,80,81,82,83].

It was found that in addition to ethanol synthesis during alcoholic fermentation, highly concentrated yeast cells also synthesize a number of metabolites [83,84] that specifically inhibit the metabolic activity of possible competitors: other types of yeast and bacteria that can contaminate the reaction medium. That is, such BCs not only provide high yields of the target product, but actually also maintain the “sterility” of the fermentation medium. The triggering of protective substance production mechanisms depends on cell density and is also regulated by QS. Thus, it was shown that the death of yeasts other than *Saccharomyces* in ethanol fermentation with mixed cultures was caused only when the cultures reached a high cell density (about 10^7^ cells/mL) [84].

It should be noted that in a number of yeast cells, more than 12 vol% ethanol accumulated in the medium leads to the complete suppression of QSMs synthesis. It has been established that with an increase in the concentration of ethanol, the rate of production of 2-phenylethanol, tryptophol, and tyrosol decreases, which is associated with a general disruption of numerous cellular processes caused by ethanol stress [85]. In light of that, regulation of the fermentation process by controlled introduction of QSMs that stimulate yeast cells to maintain high levels of ethanol production in reaction media with highly concentrated cell populations is of interest.

Therefore, the introduction of QSMs into biocatalytic systems based on yeast cells allows the formation of BCs with improved characteristics and ensures the biotechnological processes proceed effectively by increasing the cell tolerance to negative factors, including contamination of foreign microflora.

## 5. Use of Information about QS in Filamentous Fungus Cells for Their Application as Biocatalysts

Today the QS effect has been found in many representatives of filamentous fungi (*Aspergillus* spp., *Penicillium* spp., *Rhizopus* spp., *Mucor* spp., etc.) [5,86,87,88,89]. QS regulates several processes in fungi, such as physiological responses, sporulation, morphological differentiation, biofilm formation, and the biosynthesis of secondary metabolites, including mycotoxins and antibiotics [5,89,90].

The most intensive synthesis of self-protective agents in filamentous fungi was noted in the QS state and during co-cultivation with a number of bacterial cells, since it is necessary to meet their own needs for nutrients and suppress the growth of competitive microorganisms. In this regard, on the one hand, there is interest in the biotechnological production of antibiotics using filamentous fungi that are part of highly concentrated populations [91], and on the other hand, there is a question of the safety of the resulting products and the need to control residual concentrations of mycotoxins in it, in particular, in antimicrobials.

It has been established that fungi are able to produce several types of lactone-containing compounds that act as QSMs [92]. γ-butyrolactone-containing molecules such as multicolic acid function as QSMs for filamentous fungi [89]. Managing the QS allows a shift in the metabolism of BCs towards one or another target product, as well as the regulation of the population size and its resistance to the action of various compounds. As a result of managing quorum interactions, it becomes possible to support the cells of filamentous fungi in their productive state (Table 5) [5,92,93,94].

It was found that the introduction of γ-butyrolactones to the medium with *P. sclerotiorum* cells enhances the production and secretion of sclerotiorin, which is used as an antibacterial agent [92].

Farnesol affects the morphology of filamentous fungi and cellulase production by *P. decumbens* cells in the same way as in yeast. In this case, the addition of a high concentration of exogenous farnesol (1 mM) promotes the growth of hyphae and an increase in the level of the secretion of cellulases [5], which makes it attractive to use enzyme-producing BCs under such conditions.

Farnesol promotes secretion of other enzymes as well, so adding farnesol, a sesquiterpene alcohol, increases the production of laccases by *T. versicolor* and *P. sanguineus* in pineapple waste solid-state fermentation. Extracellular laccase production reaches a maximum of 77.88 ± 5.62 U/g (236% higher than control) in farnesol-induced *T. versicolor* cells on the 17th day, while under similar conditions, the maximum laccase activity of *P. sanguineus* cells was 130.95 ± 2.20 U/g (159% increase) [93].

The addition of linoleic acid as QSMs for *Aspergillus terreus* improved the production of a secondary metabolite of lovastatin, which is used to lower human blood cholesterol [94].

The introduction of γ-heptalactone isolated from the medium obtained in the stationary phase of growth of *Aspergillus nidulans* cells into media with a low concentration of the same cells reduced the lag phase of these cells to zero and led to the increased production of penicillin [5].

It is known that filamentous fungi, being in a state of highly concentrated cell populations with limited availability of nutrients, change the spectrum of hydrolytic enzymes secreted by them, in particular, by expanding the number of their isoforms. For example, under QS conditions *Mucor circinelloides* cells synthesize six isoforms of the malic enzyme (EC 1.1.1.40). One isoform (isoform IV) appearing only after the depletion of nitrogen source in the environment was associated with lipid accumulation. Isoforms I, II, V, and VI were involved in anaerobic growth and appeared only under conditions of limited O_2_ content, i.e., at increased cell concentration and high mycelium packing density. Isoform III, apparently, is constitutive and formed under conditions of active (balanced) growth [95].

A similar appearance of multiple isoforms in the spectra of endoglucanases and β-glucosidases was detected in the fungus *A. terreus*, which depended on the duration of cultivation of these cells [96]. Furthermore, an increase in the number of hydrolase isoforms in enzymatic complexes was revealed for the fungi *M. circinelloides* and *Fusarium solani*, studied in free and immobilized form [9,97].

Immobilization of the fungal producer allows increasing the synthesis of secondary metabolites, such as statins—substances with antimicrobial and antitumor activity [98].

It has been shown that the introduction of an immobilized BC based on filamentous fungi at known high concentrations into the nutrient medium makes it possible to obtain significant yields of such metabolites as valuable organic acids. At the same time, it is important to note that this approach makes it possible to obtain the expected effect on a variety of BCs when obtaining several different organic acids from a wide range of substrates with complex composition [99,100]. The use of a fungal BC in the form of highly concentrated cell populations can significantly increase the synthesis of organic acids and avoid contamination by foreign microflora. The use of immobilization and different carriers for filamentous fungi makes it possible to vary the packing density of hyphae and, thus, manage the quorum state of cells [9].

It has been established that an effective approach to increase the enzymatic activity or synthesis of metabolites in filamentous fungi in biocatalytic processes is their co-cultivation with other filamentous fungi or bacteria [101,102]. In this case, in the process of competition for a food source, filamentous fungi secrete an increased level of enzymes that allow them to use substrates that are inaccessible to other microorganisms. Microplastics are among such substrates, and therefore, filamentous fungi are considered today as BCs that can provide the application of enzymes necessary to solve the environmental problems associated with plastic micropollutants [103].

Here it is worth noting that further studies of the mechanisms and possibilities of controlling quorum interactions in populations of microscopic fungi can make it possible not only to control their biocatalytic potential, but also to direct it towards obtaining target products. For filamentous fungi, such studies are far inferior in depth and volume to those that have already been performed for bacterial cells, and therefore, there is still a huge potential in obtaining highly active BCs with adjustable properties.

## 6. Use of QS-Effectors in Development and Application of Biocatalytic Systems Based on Phototrophic Microorganisms

The use of biocatalytic systems based on populations of microalgae and cyanobacteria with an increased concentration of cells, as a rule, is concentrated in two main areas of research: synthesis of valuable components in the biomass of phototrophs (antioxidants, vitamins, lipids, etc.) [104,105], and wastewater treatment [106,107,108].

For these phototrophic microorganisms, it was found that when a certain cell density is reached in the population, their growth rate increases notably [109,110,111]. This information is actively used for the fastest possible accumulation of biomass of phototrophic microorganisms and solving various biotechnological problems with its help. It should be noted that in biotechnological processes, the maintenance of constantly high concentrations of phototrophic microorganisms in the QS state is achieved due to the fact that their growth is usually supported by the fed-batch method of cultivation, that is, no more than 10% of the volume of the medium with cells is periodically drained from the reactor, which is compensated by the addition of fresh nutrient medium.

It should be noted that with autotrophic cultivation, this approach has limitations, since with an increase in population density, the light intensity necessary for the cultivation of microalgae penetrates worse through a medium with a high density of cells, and this can slow down their growth [112,113].

The availability of light energy can become a limiting factor in the formation and use of artificial BCs based on immobilized microalgae cells [114,115], although the immobilization of phototrophic microorganisms itself has a nature-like character and simulates the conditions in which they exist in the environment. The biofilms formed in the laboratory on the basis of microalgae cells and their exopolysaccharides are one of the biotechnological examples of the use of QS of phototrophic microorganisms. It is known that immobilized catalytic systems based on individual cultures of phototrophic microorganisms differ significantly in their properties and characteristics from suspension cultures [116]. The presence of bacteria in such biocatalytic systems (if they are maintained in non-sterile conditions) promotes an increase in the rate of biofilm formation and in their thickness (Table 6) [115,117,118,119,120,121,122].

Phototrophic microorganisms as BCs for the treatment of various wastewaters, which allow not only the removal of pollutants, but also the accumulation of target components of biomass (lipids, carotenoids, PSs) in the biomass of free growing cells, attract great attention from biotechnologists and are interesting from a scientific and practical point of view (Table 7) [123,124,125,126,127,128,129,130,131,132,133]. Phototrophic microorganisms mainly exist as a part of different consortia with other microorganisms under environmental conditions. They are “ideal” candidates for mutually beneficial coexistence with aerobic microorganisms participating in biotechnological processes, since phototrophs actively consume carbon dioxide, providing “partners” with oxygen in the consortium [115,118,119].

Since sterile conditions are not maintained during the use of phototrophic microorganisms for wastewater treatment, consortia of phototrophic microorganisms and bacteria are often formed and widely used as BCs [123,128].

In the case of directed accumulation of phototrophic biomass, in order to isolate valuable products to intensify the process of biomass accumulation, and to facilitate its collection by sorption, it is often proposed to co-culture microalgae with cells of mycelial fungi or bacteria that secrete flocculating substances. It has already been established that relationships in such systems are regulated by QSMs [134]. PSs secreted by phototrophic microorganisms, whose synthesis is controlled by quorum factors, play a special role in the formation of different consortia of microalgae cells with bacteria and fungi, which are interesting as BCs for obtaining various biotechnological products [130,131,132,133,135,136].

As the main participants of aerobic natural consortia with microalgae under QS regulation, it is necessary to note the cells of the family *Xanthomonadaceae*, *Rhodobacteraceae* and *Hyphomonadaceae* possessing AHL-production and PSs-secreting functions [135].

According to AHLs add-back test, AHLs, especially those with 8-carbon sidechains (C8-HSL, 3OHC8-HSL), played an important role in aerobic sludge granulation via secreting special extracellular proteins. For aerobic consortia, a correlation was established between the concentration of intracellular ATP, the content of PSs in the composition of emerging biofilms or granules, and the presence of C8-HSL, 3OHC8-HSL, and 3OHC12-HSL molecules in the biocatalytic systems [137]. It has been established that during the functioning of the anaerobic consortia as BCs, the state of cells is constantly self-regulating [138].

Understanding the QS mechanisms by which different microorganisms interact with each other today serves as the basis for the compilation of artificial multicomponent microbial consortia as promising and effective BCs or enables the regulation of the work of self-formed multicomponent natural cell populations that are used as BCs in biotechnological processes, in particular, in water purification processes.

## 7. The Ability to Control BCs in the QS State through the Determination of Intracellular ATP Concentration

In the process of developing and using BCs, the question often arises about the need to monitor the state of the cells of microorganisms in their compositions. This is the key to the effective production and application of living cell-based BCs in various biotechnological processes. For BCs, whose functioning is associated with the formation and maintenance of the QS state in cellular populations, the determination of the intracellular concentration of ATP was found to be an effective way to control the state [139].

**Figure 2:**
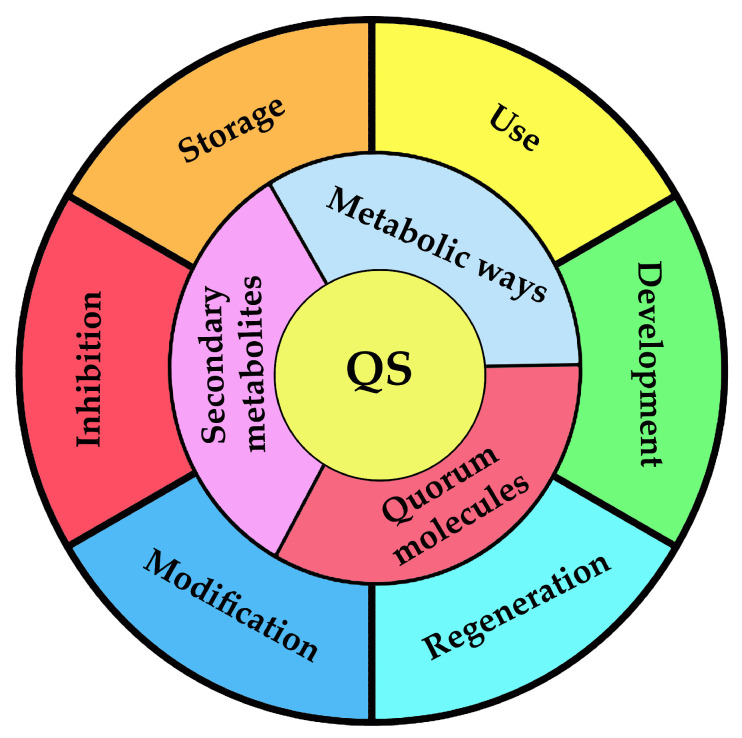
Processes with cells of microorganisms which can be monitored via measurement of intracellular ATP concentration.

ATP is the main and universal source and carrier of chemical energy in living cells for various biochemical processes, and the change in the concentration of ATP in cells occurs in the event of unfavorable conditions for their existence [140]. There is an expenditure of internal energy resources for cell survival, which is accompanied by a decrease in intracellular ATP concentration for a short period of time. By changing the content of intracellular ATP, it is possible to judge the intensity of metabolic processes occurring in the cells of microorganisms (Figure 2). It can be said that the concentration of intracellular ATP in total reflects the processes occurring in cells, and can serve as a criterion for its viability and metabolic activity. This method is appreciated for a quick and adequate analysis that allows researchers and technologists to quickly respond to the changes that occur in the cells of a variety of microorganisms [141,142,143,144]. Separately, it is necessary to note the high sensitivity of the method [139].

Since BCs, whose use is based on the maintenance of QS, are characterized, as a rule, by a high density of cell populations having, respectively, a significant total level of ATP concentration, the transition of cells in the BCs to the QS state is easy to determine by the concentration of ATP. For example, the work with an artificial consortium consisting of microalgae cells *C. vulgaris* and bacterial *Rhodococcus erythropolis* and *Pseudomonas esterophilus* cells was conducted, and it was found that the state of cells, which is characterized by a significant increase in the rate of accumulation of microalgae biomass and the rate of decomposition of organophosphorus pesticides, is associated with a sharp change in the concentration of ATP: it increased from 33 to 80 µM in suspension cells of microalgae, and it changed from 0.4 to 15.2 µM in immobilized cells [106].

A similar effect of a sharp increase in the concentration of intracellular ATP was found when selecting the ratios of jointly immobilized cells of *R. erythropolis* and *P. esterophilus* [141]. In this case, Gram-negative and Gram-positive bacterial cells were combined in the composition of the created BCs to achieve a balanced QS state inside of artificial consortium. An increase in lactase activity in this biocatalytic system, coupled with a sharp increase in the concentration of ATP in the composition of granules of BCs, confirmed the role of cellular QS in the effectiveness of such biosystems.

For aerobic sludge, a correlation was also established between the concentration of intracellular ATP, the content of exopolysaccharides in the granules and the presence of C8-HSL, 3OHC8-HSL, and 3OHC12-HSL in the medium with the biocatalytic system [133]. When studying the characteristics of a natural methanogenic consortia, usually existing in the QS state and catalyzing the production of biogas enriched with methane, and using the method of bioluminescent ATP-metry, it was found that the viability of cells in such BCs and the stability of their functioning can be effectively controlled by determining the concentration of ATP in the biosystems [145].

The research on the effect of Zn(II) on the natural ANAMMOX consortium in the QS state disclosed the negative effect of the zinc ions on cells. It was accompanied by a simultaneous decrease in the level of synthesis of AHL, dehydrogenase, nitrogen-related enzymatic activities, and ATP [146]. Thus, it is possible to control the negative impact of various factors on BCs with complex microbial compositions and take into account the possible regeneration of BCs (Figure 2).

The possibilities of using bioluminescent ATP-metry at various stages of obtaining, using, storage and regenerating BCs, which are cells of microorganisms stabilized in populations with a high density of microbial biomass, have been demonstrated [139,143,144]. Thus, it can be confidently stated that monitoring the concentration of intracellular ATP is an effective approach to obtaining and monitoring the state of BCs and their functioning, and it enables the control of BCs on the basis of the mechanisms of QS action.

## 8. Conclusions

The need to develop and use new biosystems that allow the improvement and implementation of effective biotechnological processes forms a definite scientific challenge for researchers studying BCs based on the use of microorganisms in high-density cell populations. Modern physical-chemical and biochemical methods of analysis make it possible to expand the understanding of what is the true cause of many properties manifested by cells in such BCs, to clarify or even change previously existing representations and interpretations of certain phenomena and processes, to find effective methods of managing them. This review raises questions of the importance of studying the mechanisms and molecular participants of QS, not from the point of view of suppressing this phenomenon and searching for antimicrobial agents, which is the focus of most current quorum studies, but discusses the positive biocatalytic aspects of such a state of cells, both individual cultures and biosystems with heterogeneous microbial composition.

## Figures and Tables

**Table 1:** Use of various QS systems and approaches to regulation of action efficiency of BCs based on different cell populations for improved biotechnological processes.

Regulation of QS [Reference]	Biocatalysts	Process	Product
Lux [12,13,14,15,16]	ANAMMOX bacteria	Regulation of ANAMMOX activity of biomass by *N*-acyl homoserine lactones	Nitrous oxide (N_2_O) and Molecular N_2_
Lux [17]	*Escherichia* *coli*	Synthetic QS system in the genetic circuits for the cell–cell communication among the consortia	Isopropanol
Lux, Esa [18]		QS-based activation in the system of riboregulated switchable feedback promoters (rSFPs) to regulate multiple feedback networks	Amorphadiene or Oxygenated taxane
Lux [19]		The QS circuits for diverse metabolic control	Salicylic acid and 4-hydroxycouma-rin
Esa [20]		The QS system for dynamic metabolic control	4-hydroxyphenylacetic acid
Lux [21]		Development of QS system for inducer-free production	Bisabolene
Lux, Agr [22]		Two orthogonal QS-based circuits for modulation of dynamically switching phenotypes	Medium chain fatty acids
Lux [23]		A self-induced dynamic regulated expression (SIDRE) for metabolic control	Alginate lyase and Esterase
Esa [24]		The bifunctional QS switches based on the Esa QS system	5-aminolevulinic acid
Lux [25]		The QS-metabolic toggle switch (MTS) system	Pyruvate and Acetate
Spo0A [26]	*Bacillus subtilis*	The bifunctional Phr60-Rap60-Spo0A QS system for dynamic metabolic control	Menaquinone-7
DegU [27]		Engineered DegU QS circuits for metabolic control	Poly-γ-glutamic acid
Lux [28]		A toolbox of autoinduction modules for control of gene expression	Vitamin B_2_
ComQXPA [29,30]	*B. amyloliquefaciens*	Mutation of *srfA* (Δ*srfA*—a gene cluster for biosynthesizing surfactin) influencing the carbon metabolism	Acetate
Lux [31]	*Gluconobacter* *oxydans*	The programmed cell-death module based on the QS system to reduce the competition between species	2-keto-L-gulonic acid
Las, Rhl [32]	*Pseudomonas* *aeruginosa*	The regulation of aerobic denitrification	Nitrous oxide (N_2_O) and Molecular N_2_
RRNPP [33]	*Clostridium* *saccharoperbutylacetonicum*	The regulation of anaerobic acetone– butanol–ethanol fermentation	Organic solvents

**Table 2:** Production of polysaccharides by BCs based on immobilized cells in state of QS.

Polysaccharides	Microorganism, Initial Cell Concentration	Product Concentration
Dextran [45]	*Leuconostoc mesenteroides* immobilized in poly(vinyl alcohol) (PVA) cryogel, 0.2 g (dry weight)/L	Immobilized cells— 41.0 g/L, Free cells—34.0 g/L
Pullulan [46]	*Aureobasidium pullulans* immobilized in PVA cryogel, 7.5 g (dry weight)/L	Immobilized cells— 10.0 g/L, Free cells—6.0 g/L
Bacterial cellulose [47]	*Komagataeibacter xylinum* immobilized in PVA cryogel, 0.03 g dry weight/L	Immobilized cells— 2.9 g/L, Free cells—1.8 g/L
Bacterial cellulose [48]	*Gluconacetobacter kombuchae* immobilized on loofa sponge, 5% *v/v*	Immobilized cells— 15.5 g/L, Free cells—7.4 g/L
Alginate [49]	*Azotobacter vinelandii* immobilized in agar microporous membrane, 2.25 mg biomass (dry wt.)/mL of gel	Immobilized cells— 0.9 g/L, Free cells—1.0 g/L
Xanthan [50]	*Xanthomonas campestris* immobilized on polyurethane foam, 1 cube with immobilized cells (10^5^ colony forming unit/mL) in 9 mL of medium	Immobilized cells— 59.9 g/L, Free cells—16.0 g/L
Xanthan [51]	*X. campestris* immobilized on polyethylene, OD_600_—0.25–0.26	Immobilized cells— 8.0 g/L, Free cells—3.0 g/L
Xanthan [52]	*X. campestris* immobilized in calcium alginate-polyvinyl alcohol-boric acid beads, 5% (*v*/*v*)	Immobilized cells— 9.2 g/L, Free cells—6.5 g/L
Levan [53]	*Zymomonas mobilis* immobilized on loofa (0.19 g/L); *Z. mobilis* immobilized on sugarcane bagasse (0.37 g/L)	Immobilized cells on loofa—19.0 g/L Immobilized cells on bagasse—10.0 g/L
Fructo- oligosaccharides [54]	*A. pullulans* immobilized on reticulated polyurethane foam, 9 × 10^7^ spores/mL	Immobilized cells— 108.2 g/L Free cells—92.1 g/L
Curdlan [55]	*Agrobacterium* sp. immobilized on loofa sponge, 500 mg/L of the lyophilized biomass	Immobilized cells— 17.8 g/L Free cells—14.1 g/L
Succinoglycan [56]	*Agrobacterium radiobacter* immobilized on loofa sponge, 300 mg/L of lyophilized cell biomass	Immobilized cells— 14.0 g/L Free cells—11.7 g/L
Exopolysaccharides [57]	*Lactobacillus delbrueckii* immobilized on polyurethane foam, 5% *w/v* of the immobilized cells	Immobilized cells— 562.8 mg/L Free cells—583.7 mg/L
Exopolysaccharides [58]	*L. plantarum* immobilized in agar gel, 3–5% *w*/*v*	Immobilized cells— 1489.9 mg/L Free cells—1513.1 mg/L

**Table 3:** Application of QSMs to regulate the population density of yeast cells in various processes.

Strain; Concentration of QSMs [Reference]	Process
*C. tropicalis*; phenylalanine (2 g/L) as inductor for synthesis of phenylethanol (0.25–0.30 g/L) [60]	Wastewater treatment
*Candida tropicalis*; 2,4-di-tert-butylphenol (100 μM) [61]	Oil refinery wastewater treatment
*Torulaspora delbrueckii*; 2-phenylethanol (226 mg/L) [62]	Production of honey with enhanced aroma and flavor profile
*S. cerevisiae*, immobilized in Ca-alginate gel; 2-phenylethanol (0.2% *w*/*v*) [63]	Ethanol fermentation of algal biomass hydrolysate
*S. cerevisiae*; 2-phenylethanol (0.12 g/L) [64]	Ethanol fermentation and decrease in media contamination by *Lactobacillus plantarum*
*S. cerevisiae*; 2-phenylethanol, tryptophol and tyrosol (0–0.2% *w*/*v*) [65]	Ethanol fermentation
*S. cerevisiae, Wickerhamomyces anomalus,* *Candida glabrata* and *C. tropicalis;* tyrosol (50 µM) [66]	Ethanol fermentation
*S. cerevisiae, T. delbrueckii,* *Metschnikowia pulcherrima,* *Hanseniaspora uvarum* and *Starmerella bacillaris*; tryptophol and melatonin (0.5–1.0 g/L) [67]	Ethanol fermentation
*S. cerevisiae*; 2-phenylethanol and tryptophol (10 μM) [68]	Biofilm augmentation in yeast-based microbial fuel cells
*Wickerhamomyces* anomalus, *S. cerevisiae,* *C. tropicalis* and *C. glabrata;* tyrosol (191.5–617.2 µM) [69]	Enhance heavy metals tolerance during brewed fermentation
*Debaryomyces nepalensis;* 2-phenylethanol (2 mmol/L) [70]	Enhance fruit preservation
*Pichia kudriavzevii*; 2-phenylethanol (6 mg/L) [71]	Baijiu fermentation
*S. cerevisiae*; acetic acid as a QSM (1.5 g/L) [72]	2,3-Butanediol production in co-fermentation with *Acetobacter pasteurianus*

**Table 4:** Ethanol fermentation catalyzed by BCs in the form of immobilized concentrated cells.

Cells/Carrier	Conditions and Substrate	Concentration of Ethanol/Number of Cycles
*S. cerevisiae* immobilized into calcium alginate-polyvinyl alcohol gel beads [79]	*Madhuca indica* (Mahua flowers) (reducing sugars—150 g/L) pH 5.5, 30 °C, 16 h,	Batch fermentation: Free cells—56.0 g/L Immobilized cells—67.8 g/L Reuse of immobilized cells in 5 cycles
*S. cerevisiae* immobilized on delignified sugarcane bagasse [80]	pH 4.8, 30 °C, 48 h Diluted molasses (231 g/L of total sugar) 10% (*v*/*v*) of the starter culture (OD_600_ approximately 1.0)	Batch fermentation–Immobilized cells—194.3 g/L Free cell—176.6 g/L Reuse of immobilized cells in 5 cycles
*S. diaststicus* immobilized in *Mucuna urens* matrix cross-linked with glutaric aldehyde [81]	pH 4.5, 30 °C, 72 h Corn straw (10%), 10% (*v*/*v*) of the starter culture	Immobilized cells—24.9 g/L, Free cell—15.4 g/L Reuse of immobilized cells in 4 cycles
*S. cerevisiae* immobilized on cotton [82]	pH 5.6, 36 °C, 24 h Corn stalks juice, 15% (*w*/*v*) immobilized yeast cell	Immobilized cells—62.1 g/L, Free cell—47.4 g/L Reuse of immobilized cells in 3 cycles
*Pichia kudriavzevii* immobilized on corncobs [83]	42 °C, 40 h Yeast Extract–Peptone–Dextrose medium (Glucose—100 g/L) initial cell concentration— 4 × 10^7^ cells/mL	Immobilized cells—44.5 g/L, Free cell—42.5 g/L Reuse of immobilized cells in 4 cycles

**Table 5:** Use of various QSMs to improve efficiency of action of BCs based on filamentous fungi.

Biocatalysts [Reference]	QSMs Used for Regulation	Product with Increased Level of Synthesis
*Penicillium sclerotiorum* [92]	γ-Butyrolactone	Sclerotiorin
*P. decumbens* [5]	Farnesol	Cellulases
*Trametes versicolor,* *Pycnoporus sanguineus* [93]	Farnesol	Laccases
*Aspergillus terreus* [94]	Linoleic acid	Lavostatin

**Table 6:** Effect resulting from the use of various immobilized BCs with phototrophic microorganisms.

Biocatalysts [Reference]	Cell Concentrations	Effect
*C. vulgaris* immobilized in Ca-alginate capsules [117]	100 g dry matter/L *C. vulgaris*	High efficiency of CO_2_ biosequestration, high growth rate of microalgae biomass
*Pseudomonas putida* and *C. vulgaris* co-immobilized in Ca-alginate gel beads [118]	5 × 10^7^ cells/mL *C. vulgaris* 5 × 10^8^ cells/mL *P. putida*	Increase in growth rate of both microorganisms, symbiotic interactions between cells, quick removal of nutrients
Co-culture of suspended *P. putida* and *C. vulgaris* immobilized in Ca-alginate gel [119]	5 × 10^6^ cells/mL *C. vulgaris* 2.5 × 10^6^ cells/mL *P. putida*	Quick removal of nutrients and chemical oxygen demand (COD), enhanced growth rate of both microorganisms, symbiotic interactions between cells
*Scenedesmus obliquus*, *C. vulgaris* and *Chlorella sorokiniana* co-immobilized with bacterial sludge in Ca-alginate gel beads [115]	The volumetric ratio of sodium alginate: microalgae concentrate: sludge bacterial concentrate was 8:1:1. Microalgae and bacterial sludge were concentrates up to 32.0 g total suspended solids/L for each.	Algal growth increased by 2.25 times as compared to suspended microalgae cells, removal of COD and total nitrogen content improved from 78.5 to 82.9% and from 68.5 to 84.4%, respectively
*C.vulgaris* f. *globosa* immobilized on analcime-bearing rock [120]	The formed microalgae biofilm contained 1.6 × 10^4^ cells/mm^2^	Considerable increase in phenol removal (from 27 to 93%)
*Nannochloropsis* sp. immobilized on Ca-alginate gel beads [121]	0.2 g/L *Nannochloropsis* sp. in the carrier	More intensive biomass accumulation (1.27 g/L) and COD reduction (71%) as compared to suspended free cells which accumulated 0.37 g/L biomass and provided 48% reduction in COD
*C. sorokiniana* immobilized on modified calcined mussel shell powder [122]	0.25 × 10^6^ cell/mL *C. sorokiniana*	Quick increase in biomass concentration by 10 times, high removal rate of N (95.0%) and P (88.6%)

**Table 7:** Application of various phototrophic microorganisms as BCs under QS control.

Biocatalysts [Reference]	Application of BCs	Effect under QS Control
Microalgal-bacterial aerobic granular sludge [123]	Wastewater treatment	Formation of biofilm
*Chlorophyta* sp. [124]	Accumulation of lipids	QSMs provoke 84% increase in lipid content of microalgae biomass
*Chlorella* sp., *Scenedesmus* sp. [125]	Accumulation of biomass and lipids	Co-culture cultivation reduced growth inhibition by 50%. Synthesis of C6-HSL and stimulation of lipid productivity (20–79% higher as compare to monocultures)
*C. sorokiniana* [126]	Accumulation of biomass and lipids	QSMs enhanced the algal biomass productivity and lipid content (they increased by 2.25 and 1.28 times, respectively)
*Tetraselmis striata* and *Pelagibaca bermudensis* [127]	Accumulation of biomass and lipids	Biomass production in a saline/marine medium at a broad range of pH, salinity, and temperature/light conditions, as well as nutrient limitation with a 1.2–3.6-fold growth rate increase
*Chlorella* sp. and bacteria from active sludge [128]	Accumulation of PSs	Molecules of 12-AHL stimulated synthesis of PSs and formation of flocs
*C. vulgaris* [129]	Phenol degradation	Biofilm formation
Consortia *Aspergillus fumigatus* and *Synechocystis* sp. [130]	Treatment of wastewaters with heavy metals	Increased accumulation in living microalgae biomass by adhesion on biomass of filamentous fungi and good wastewater treatment
*Chlorella* sp. and *Aspergillus* sp. [131]	Oil-rich biomass production and molasses wastewater treatment	Increased efficiency of water treatment and lipid accumulation in the biomass
*Rhodotorula glutinis* and *C. vulgaris* [132]	Accumulation of carotenoids and wastewater purification	Increased accumulation of carotenoids as antioxidants
Native mixture of phototrophic microorganisms, *Trichoderma reesei*, and *A. niger* [133]	Ethanol production	Increased synthesis and secretion of cellulases

## Data Availability

Not applicable.
